# Supplementary material for: Factors associated with unintended pregnancy and contraceptive practices in justice-involved adolescent girls in Australia
Source: PLoS One. 2024 Jun 18;19(6):e0304825. doi: 10.1371/journal.pone.0304825 (PMC11185493; doi:10.1371/journal.pone.0304825)
Supplement: S1 File — (DOCX) [file pone.0304825.s001.docx]

**MEH-JOSH SURVEY**

**TELEPHONE INTERVIEW QUESTIONS**

[**SURVEY DETAILS** 2](#_Toc492997611)

[**AGE CHECK** 2](#_Toc492997612)

[**PAST CONTACT WITH THE CRIMINAL JUSTICE SYSTEM CHECK** 2](#_Toc492997613)

[**SOCIODEMOGRAPHICS** 3](#_Toc492997614)

[**SF1 & K10** 13](#_Toc492997615)

[**HEAD INJURY** 17](#_Toc492997616)

[**SELF-HARM (SMHAC 2014)** 19](#_Toc492997617)

[**SUICIDE (SMHAC 2014)** 21](#_Toc492997618)

[**MAJOR DEPRESSION (MINI KID 6.0)** 23](#_Toc492997619)

[**PSYCHOSIS SCREENER (SMHAC 2014)** 26](#_Toc492997620)

[**ATTENTION DEFICIT HYPERACTIVITY DISORDER (MINI KID 6.0)** 27](#_Toc492997621)

[**POST-TRAUMATIC STRESS DISORDER (MINI KID 6.0)** 29](#_Toc492997622)

[**MENTAL HEALTH SERVICE UTILISATION (SMHAC 2014)** 31](#_Toc492997623)

[**REPRODUCTIVE HEALTH (GIRLS ONLY)** 32](#_Toc492997624)

[**FATHERS AND CHILDREN (BOYS ONLY)** 39](#_Toc492997625)

[**SEXUAL IDENTITY, ATTRACTION AND SEXUAL HISTORY** 41](#_Toc492997626)

[**PERCEPTIONS OF RISK** 54](#_Toc492997627)

[**KNOWLEDGE OF HIV, STIs, HEPATITIS** 55](#_Toc492997628)

[**SEXUAL HEALTH EDUCATION AND INFORMATION** 56](#_Toc492997629)

[**HPV VACCINATIONS** 58](#_Toc492997630)

[**STI, HIV, HCV TESTING AND DIAGNOSIS** 59](#_Toc492997631)

[**ALCOHOL DEPENDENCE/ABUSE (SMHAC2014 & MINI KID 6.0)** 61](#_Toc492997632)

[**DRUG USE** 65](#_Toc492997633)

[**INJECTING DRUG USE** 67](#_Toc492997634)

[**TOBACCO (SMHAC 2014)** 69](#_Toc492997635)

[**SURVEY FEEDBACK** 71](#_Toc492997636)

[**TELEPHONE INTERVIEWER NOTES** 72](#_Toc492997637)

**MEH-JOSH QUESTIONNAIRE (COMPUTER)**

| SURVEY DETAILS | |
| --- | --- |
| **ID.** UNIQUE ID | **Type in answer** |
| **INTERVIEWER.** INTERVIEWER NAME | ***Type in name*** |
| **DATE.** TODAY'S DATE (DD/MM/YYYY) | ***DD/MM/YYYY*** |
| **STATE.** AUSTRALIAN STATE (QLD or WA) | ***Type in answer*** |
| **PLACE.** PLACE OF INTERVIEW | ***Type in answer*** |
| **NO.** INTERVIEW NUMBER | ***Type in answer*** |
| **INIT.** PARTICIPANT INITIALS (e.g. JD) | ***Type in answer*** |
| **We need to ask you a few questions to double check and make sure that you are right for our survey.** | |
| AGE CHECK | |
| **DOB.** Can I ask the date you were born? (DD/MM/YYYY) | ***DD/MM/YYYY*** |
| **AGECHECK.** **Computer calculated respondent's age as:** | **Value calculated by the computer** |
| **AGE.** How old are you? (Check birthdate if it does not add up to computer calculation above) | **Drop down list**  **14 years**  **15 years**  **16 years**  **17 years**  **13 YEARS OR LESS --> SAVE FILE & END SURVEY**  **18 YEARS OR OVER --> SAVE FILE & END SURVEY** |
| PAST CONTACT WITH THE CRIMINAL JUSTICE SYSTEM CHECK | |
| **HAVE YOU EVER BEEN:** | |
| **FINED.** Fined? If YES, what was your most serious offence? | ***Type in answer*** |
| **BOND.** Put on a good behaviour bond? If YES, what was your most serious offence? | ***Type in answer*** |
| **PROBATION.** Put on probation? If YES, what was your most serious offence? | ***Type in answer*** |
| **COMMORD.** Placed on a community order? If YES, what was your most serious offence? | ***Type in answer*** |
| **JUVDET.** Placed in juvenile detention? If YES, what was your most serious offence? | ***Type in answer*** |
| **PRISON.** Placed in an adult prison? If YES, what was your most serious offence? | ***Type in answer*** |
| **OTHER.** Have you ever had any other type of contact with the criminal justice system? (Example: Warnings, Caution, Curfew, Watchouse, Court) If YES, what was your most serious offence for each type of contact? | ***Specify type of contact with the criminal justice system and reason for contact*** |
| **NONE.** None of the above (SAVE FILE and END SURVEY) | **Drop down list**  **None of the above --> SAVE FILE & END SURVEY** |
| **CURRENT.** Are you currently on a good behaviour bond, probation, juvenile detention, prison or on community orders or other? | **Drop down list**  **1=Yes, in Juvenile detention**  **2=Yes, In Prison**  **3=Yes, On community orders**  **4=Yes, On probation**  **5=Yes, On a good behaviour bond**  **6=Yes, Other**  **7=None of the above** |

| SOCIODEMOGRAPHICS | |
| --- | --- |
| **Let us start, we would like to first ask you a few questions about yourself.** | |
| **HOME.** What town or suburb or postcode do you live in now? Or are you currently homeless? (If 'homeless', type 'homeless') | ***Type in answer*** |
| **GENDER.** Are you male or female? | **Drop down list**  **1=Male**  **2=Female**  **3=Transgender** |
| **POB.** Were you born in Australia? If you were not born in Australia, please tell me where? | **Drop down list**  **Australia**  **New Zealand**  **United Kingdom**  **Don't know/Unsure**  **Prefer not to say**  **MORE...**  **Afghanistan**  **Albania**  **Algeria**  **American Samoa**  **Andorra**  **Angola**  **Anguilla**  **Antarctica**  **Antigua and Barbuda**  **Argentina**  **Armenia**  **Aruba**  **Austria**  **Azerbaijan**  **Bahamas, The**  **Bahrain**  **Bangladesh**  **Barbados**  **Belarus**  **Belgium**  **Belize**  **Benin**  **Bermuda**  **Bhutan**  **Bolivia**  **Bosnia and Herzegovina**  **Botswana**  **Bouvet Island**  **Brazil**  **British Indian Ocean Territory**  **British Virgin Islands**  **Brunei**  **Bulgaria**  **Burkina Faso**  **Burma**  **Burundi**  **Cambodia**  **Cameroon**  **Canada**  **Cape Verde**  **Cayman Islands**  **Central African Republic**  **Chad**  **Chile**  **China**  **Christmas Island**  **Cocos (Keeling) Islands**  **Colombia**  **Comoros**  **Congo, Democratic Republic of the**  **Congo, Republic of the**  **Cook Islands**  **Costa Rica**  **Cote d'Ivoire**  **Croatia**  **Cuba**  **Curacao**  **Cyprus**  **Czech Republic**  **Denmark**  **Djibouti**  **Dominica**  **Dominican Republic**  **Ecuador**  **Egypt**  **El Salvador**  **Equatorial Guinea**  **Eritrea**  **Estonia**  **Ethiopia**  **Falkland Islands (Islas Malvinas)**  **Faroe Islands**  **Fiji**  **Finland**  **France**  **France, Metropolitan**  **French Guiana**  **French Polynesia**  **French Southern and Antarctic Lands**  **Gabon**  **Gambia, The**  **Gaza Strip**  **Georgia**  **Germany**  **Ghana**  **Gibraltar**  **Greece**  **Greenland**  **Grenada**  **Guadeloupe**  **Guam**  **Guatemala**  **Guernsey**  **Guinea**  **Guinea**  **Guyana**  **Haiti**  **Heard Island and McDonald Islands**  **Holy See (Vatican City)**  **Honduras**  **Hong Kong**  **Hungary**  **Iceland**  **India**  **Indonesia**  **Iran**  **Iraq**  **Ireland**  **Isle of Man**  **Israel**  **Italy**  **Jamaica**  **Japan**  **Jersey**  **Jordan**  **Kazakhstan**  **Kenya**  **Kiribati**  **Korea, North**  **Korea, South**  **Kosovo**  **Kuwait**  **Kyrgyzstan**  **Laos**  **Latvia**  **Lebanon**  **Lesotho**  **Liberia**  **Libya**  **Liechtenstein**  **Lithuania**  **Luxembourg**  **Macau**  **Macedonia**  **Madagascar**  **Malawi**  **Malaysia**  **Maldives**  **Mali**  **Malta**  **Marshall Islands**  **Martinique**  **Mauritania**  **Mauritius**  **Mayotte**  **Mexico**  **Micronesia, Federated States of**  **Moldova**  **Monaco**  **Mongolia**  **Montenegro**  **Montserrat**  **Morocco**  **Mozambique**  **Namibia**  **Nauru**  **Nepal**  **Netherlands**  **New Caledonia**  **Nicaragua**  **Niger**  **Nigeria**  **Niue**  **Norfolk Island**  **Northern Mariana Islands**  **Norway**  **Oman**  **Pakistan**  **Palau**  **Panama**  **Papua New Guinea**  **Paraguay**  **Peru**  **Philippines**  **Pitcairn Islands**  **Poland**  **Portugal**  **Puerto Rico**  **Qatar**  **Reunion**  **Romania**  **Russia**  **Rwanda**  **Saint Barthelemy**  **Saint Helena, Ascension,**  **and Tristan da Cunha**  **Saint Kitts and Nevis**  **Saint Lucia**  **Saint Martin**  **Saint Pierre and Miquelon**  **Saint Vincent and the Grenadines**  **Samoa**  **San Marino**  **Sao Tome and Principe**  **Saudi Arabia**  **Senegal**  **Serbia**  **Seychelles**  **Sierra Leone**  **Singapore**  **Sint Maarten**  **Slovakia**  **Slovenia**  **Solomon Islands**  **Somalia**  **South Africa**  **South Georgia and the Islands**  **South Sudan**  **Spain**  **Sri Lanka**  **Sudan**  **Suriname**  **Svalbard**  **Swaziland**  **Sweden**  **Switzerland**  **Syria**  **Taiwan**  **Tajikistan**  **Tanzania**  **Thailand**  **Timor**  **Togo**  **Tokelau**  **Tonga**  **Trinidad and Tobago**  **Tunisia**  **Turkey**  **Turkmenistan**  **Turks and Caicos Islands**  **Tuvalu**  **Uganda**  **Ukraine**  **United Arab Emirates**  **United States**  **United States Minor Outlying Islands**  **Uruguay**  **Uzbekistan**  **Vanuatu**  **Venezuela**  **Vietnam**  **Virgin Islands**  **Wallis and Futuna**  **West Bank**  **Western Sahara**  **Yemen**  **Zambia**  **Zimbabwe**  **Zambia**  **Zimbabwe** |
| **ATSI**. Are you Aboriginal or Torres Strait Islander, both or neither? | **Drop down list**  **1 = Aboriginal**  **2 = Torres Strait Islander**  **3 = Both Aboriginal and Torres Strait Islander**  **4 = Neither Aboriginal nor Torres Strait Islander**  **9 = Prefer not to say** |
| **MARITAL.** Are you single/never married, are you living with a partner, are you in a relationship but not living with a partner, or are you married, separated or divorced? | **Drop down list**  **1 = Single/Never married**  **2 = Living with partner in the same residence**  **3 = In a relationship but not living with him/her**  **4 = Separated**  **5 = Married**  **6 = Separated/Divorced**  **7 = Widowed**  **9 = Prefer not to say** |
| **JUVENILE DETENTION HISTORY** |  |
| **JUV.** Have you ever been in JUVENILE DETENTION? | **Drop down list**  **0 = No (GO TO COM1)**  **1 = Yes (CONTINUE)**  **9 = Prefer not to say (GO TO COM1)** |
| **JUVa.** In your lifetime, how many times have you been in juvenile detention? | **Drop down list**  **1= First time/one time only/this time only**  **2 = 2 times**  **3 = 3 times**  **4 = 4 times**  **5 = 5 times**  **6 = 6 to 10 times**  **7 = More than 10 times**  **9 = Prefer not to say** |
| **JUVb.** Thinking about THIS TIME/LAST TIME you went to a juvenile detention centre, what was the most serious offence which caused you to be sent there? | ***Type in answer*** |
| **COMMUNITY ORDERS HISTORY** |  |
| **COM1.** Have you ever been on COMMUNITY ORDERS? | **Drop down list**  **0=No (GO TO EDU1)**  **1=Yes (CONTINUE)**  **9 = Prefer not to say (GO TO EDU1)** |
| **COM1a.** How many times have you been on COMMUNITY ORDERS (includes the current community order)? | **Drop down list**  **0 =Never**  **1 = Once only**  **2 = 2 times**  **3 = 3 times**  **4 = 4 times**  **5 = 5 times**  **6 = 6 to 10 times**  **7 = More than 10 times**  **9 = Prefer not to say** |
| **COM1b.** Thinking about THIS TIME/LAST TIME you went on community orders, what was the most serious offence which caused you to be sent on a community order? | ***Type in answer*** |
| **EDUCATION** |  |
| **Now, we would now like you to answer some questions about going to school.** | |
| **EDU1.** Do you go to school? (IF NO, GO TO ‘NOT AT SCHOOL’ NOEDU1) | **Drop down list**  **0 = Not at school (GO TO NOEDU1)**  **1 = At school (CONTINUE)**  **9 = Prefer not to say (NEXT MODULE)** |
| **EDU1a.** What year are you in at school? | **Drop down list**  **Year 1**  **Year 2**  **Year 3**  **Year 4**  **Year 5**  **Year 6**  **Year 7**  **Year 8**  **Year 9**  **Year 10**  **Year 11**  **Year 12**  **99 = Prefer not to say** |
| **NOT AT SCHOOL** |  |
| **NOEDU1.** What CLASS/YEAR were you in when you left school? | **Drop down list**  **Year 1**  **Year 2**  **Year 3**  **Year 4**  **Year 5**  **Year 6**  **Year 7**  **Year 8**  **Year 9**  **Year 10**  **Year 11**  **Year 12**  **99 = Prefer not to say** |
| **NOEDU1a.** What is the highest level of education that you have completed? | **Drop down list**  **1 = I did not complete primary school**  **2 = I completed primary school only**  **3 = I left school before finishing Year 10**  **4 = I completed Year 10**  **5 = I completed Year 12**  **6 = I completed technical trade qualification**  **7 = I completed a diploma or university degree**  **9 = Prefer not to say** |
| **NOEDU1b.** Which of the following best describes your occupation status now? | **Drop down list**  **1 = Employed full-time**  **2 = Employed part-time [INCLUDE Casually employed]**  **3 = Home duties**  **4 = Unemployed**  **5 = Student**  **6 = Permanently ill or unable to work**  **7 = Other**  **9 = Prefer not to say** |
| **NOEDU1c.** Are you receiving any allowances or benefits? What allowances or benefits were you receiving? | **Drop down list**  **0 = None**  **1 = Youth allowance**  **2 = Newstart**  **3 = Disability**  **4 = Other**  **9 = Prefer not to say** |

| SF1 & K10 | |
| --- | --- |
| ***These next questions are about your physical health and your state of mental health and wellbeing.*** | |
| **SF1**. In general, would you say your health is excellent, very good, good, fair or poor? | **Drop down list**  **1 = Excellent**  **2 = Very good**  **3 = Good**  **4 = Fair**  **5 = Poor**  **9 = Prefer not to say** |
| ***Next, the following questions ask about how you have been feeling in the LAST 4 WEEKS.*** | |
| **K10A**. In the last four weeks, about how often did you feel tired out for no good reason? Is that none of the time, a little of the time, some of the time, most of the time or all the time? | **Drop down list**  **1 - None of the time**  **2 - A little of the time**  **3 - Some of the time**  **4 - Most of the time**  **5 - All of the time**  **9 = Prefer not to say** |
| **K10B.** In the last four weeks, about how often did you feel nervous? Is that none of the time, a little of the time, some of the time, most of the time or all the time? |  |
| **K10C**. In the last four weeks, about how often did you feel so nervous that nothing could calm you down? Is that none of the time, a little of the time, some of the time, most of the time or all the time? |  |
| **K10D.** In the last 4 weeks, about how often did you feel hopeless? |  |
| **K10E.** In the last 4 weeks, about how often did you feel restless or fidgety? |  |
| **K10F**. In the last 4 weeks, about how often did you feel so restless you could not sit still? |  |
| **K10G.** In the last 4 weeks, about how often did you feel depressed? |  |
| **K10H.** In the last 4 weeks, about how often did you feel that everything was an effort? |  |
| **K10I.** In the last 4 weeks, about how often did you feel so sad that nothing could cheer you up? |  |
| **K10J.** In the last 4 weeks, about how often did you feel worthless? |  |

| **STRENGTHS AND DIFFICULTIES (SMHAC 2014)** | |
| --- | --- |
| **The next questions are on the basis of how things have been for you over the LAST SIX MONTHS. For each item, please tell us if it is TRUE OR NOT TRUE.** | |
| **SDQ1.** In the last six months, you try to be nice to other people. You care about their feelings | **Drop Down List**  **1 = Not True**  **2 = Somewhat True**  **3 = Certainly True**  **9 = Prefer not to say** |
| **SDQ2.** You are restless, you cannot stay still for long |  |
| **SDQ3.** You get a lot of headaches, stomach-aches, or sickness |  |
| **SDQ4.** You usually share with others, for example CDs, games, food |  |
| **SDQ5.** You get very angry and often lose your temper |  |
| **SDQ6.** You would rather be alone than with people of your age |  |
| **SDQ7.** You usually do as you are told |  |
| **SDQ8.** You worry a lot |  |
| **SDQ9.** You are helpful if someone is hurt, upset or feeling ill |  |
| **SDQ10.** You are constantly fidgeting or squirming |  |
| **SDQ11.** You have one good friend or more |  |
| **SDQ12.** You fight a lot. You can make other people do what you want. |  |
| **SDQ13.** You are often unhappy, depressed or tearful |  |
| **SDQ14.** Other people your age generally like you |  |
| **SDQ15.** You are easily distracted, you find it difficult to concentrate |  |
| **SDQ16.** You are nervous in new situations. You easily lose confidence |  |
| **SDQ17.** You are kind to younger children |  |
| **SDQ18.** You are often accused of lying or cheating |  |
| **SDQ19.** Other children or young people pick on you or bully you |  |
| **SDQ20.** You often volunteer to help others (parents, teachers, children) |  |
| **SDQ21.** You think before you do things |  |
| **SDQ22.** You take things that are not yours from home, school or elsewhere |  |
| **SDQ23.** You get along better with adults than with people your own age |  |
| **SDQ24.** You have many fears, you are easily scared |  |
| **SDQ25.** You finish the work you're doing. Your attention is good |  |
| **Please answer YES or NO for the next questions.** |  |
| **SDQ26.** Does your family complain about you having problems with overactivity or poor concentration? | **Drop Down List**  **1= No**  **2 = Yes, a little**  **3 = Yes, a lot**  **9 = Prefer not to say** |
| **SDQ27.** Do your teachers complain about you having problems with overactivity or poor concentration? | **Drop Down List**  **1= No**  **2 = Yes, a little**  **3 = Yes, a lot**  **9 = Prefer not to say** |
| **SDQ28.** Does your family complain about you being awkward or troublesome? | **Drop Down List**  **1= No**  **2 = Yes, a little**  **3 = Yes, a lot**  **9 = Prefer not to say** |
| **SDQ29.** Do your teachers complain about you being awkward or troublesome? | **Drop Down List**  **1= No**  **2 = Yes, a little**  **3 = Yes, a lot**  **9 = Prefer not to say** |
| **SDQ30.** Overall, do you think that you have difficulties in any of the following areas: emotions, concentration, behaviour or being able to get along with other people? | **Drop Down List**  **0 = No (GO TO NEXT MODULE)**  **1 = Yes – minor difficulties**  **2 = Yes – definite difficulties**  **3 = Yes – severe difficulties**  **9 = Prefer not to say (GO TO NEXT MODULE)** |
| **SDQ30a.** How long have these difficulties been present? Is that less than a month, 1-5 months, 6-12 months, or over a year? | **Drop Down List**  **1 = Less than a month**  **2 = 1-5 months**  **3 = 6-12 months**  **4 = Over a year**  **9 = Prefer not to say** |
| **SDQ30b.** Do the difficulties upset or distress you? | **Drop Down List**  **1 = Not at all**  **2 = Yes, a little**  **3 = Yes, a medium amount**  **4 = Yes, a great deal**  **9 = Prefer not to say** |
| **SDQ30c.** Do the difficulties interfere with your everyday life in the following areas:  ***SDQ30c1.*** *Home life?*  ***SDQ30c2.*** *Friendships?*  ***SDQ30c3.*** *Classroom learning?*  ***SDQ30c4.*** *Leisure activities?* | **Drop Down List**  **1 = Not at all**  **2 = Yes, a little**  **3 = Yes, a medium amount**  **4 = Yes, a great deal**  **9 = Prefer not to say** |
| **SDQ30d.** Do the difficulties make it harder for those around you (family, friends, teachers, etc.)? | **Drop Down List**  **1 = Not at all**  **2 = Yes, a little**  **3 = Yes, a medium amount**  **4 = Yes, a great deal**  **9 = Prefer not to say** |

| HEAD INJURY | |
| --- | --- |
| ***Next, a few questions about times you may have received an injury to your head.*** | |
| **HEAD1.** Have you ever had a head injury where you became unconscious or “blacked out”? | **Drop down list**  **0=No (GO TO YRB34)**  **1=Yes**  **9 = Prefer not to say (GO TO YRB34)** |
| **a.** How many TIMES has this happened? | **Drop down list**  **1 = Once**  **2 = Twice**  **3 = Three times**  **4 = More than three times**  **9 = Prefer not to say** |
| **b.** What caused you to become unconscious? | **Drop down list**  **1 = Motor vehicle accident**  **2 = Hit in a fight**  **3= Assault/someone hit you**  **4 = Slip and fall**  **5 = Sports injury**  **6 = Stroke**  **7 = Bullet wound**  **8 = Other**  **9 = Prefer not to say** |
| **c.** For how long were you unconscious? | **Drop down list**  **1 = Only a brief moment**  **2 = Between 10 =30 minutes**  **3 = Between 30 minutes – 24 hours**  **4 = More than 24 hours**  **8 = Don't Know**  **9 = Prefer not to say** |
| **d.** When did this occur? | **Drop down list**  **1 = Within last week**  **2 = 1 = 4 Weeks ago**  **3 = 1 = 6 Months ago**  **4 = Over 6 months and less than 2 years ago**  **5 = 2 Years ago or more**  **8 = Don’t know**  **9 = Prefer not to say** |
| **e.** Did you go to the hospital? | **Drop down list**  **0=No**  **1=Yes**  **9= Prefer not to say** |
| **HEAD2.** Did you have any physical or mental health problems as a result of these head injuries? **If Yes,** what were they? **(May have more than one answer. Select all that apply.)** | **Drop down list**  **1 = No problems**  **2 = Weakness in any part of the body**  **3 = Poor concentration**  **4 = Memory loss**  **5 = Problems finding the right words when speaking**  **6 = Problem with coordination/balance**  **7 = Personality/behavioural changes**  **8 = Anxiety or depression**  **9 = Headache**  **10 = Other**  **99 = Prefer not to say** |
| **HEAD3.** Which of these effects have not gone away (resolved)? **(May have more than one answer. Select all that apply)** | **Drop down list**  **1 = No problems**  **2 = Weakness in any part of the body**  **3 = Poor concentration**  **4 = Memory loss**  **5 = Problems finding the right words when speaking**  **6 = Problem with coordination/balance**  **7 = Personality/behavioural changes**  **8 = Anxiety or depression**  **9 = Headache**  **10 = Other**  **99 = Prefer not to say** |

| SELF-HARM (SMHAC 2014) | |
| --- | --- |
| ***These next questions ask about deliberate self-harm, that is deliberately hurting or injuring yourself without trying to end your life.*** | |
| **DELIBERATELY HURT OR INJURED SELF** |  |
| **YRB34.** Have you ever deliberately done something to yourself to cause harm or injury, without intending to end your own life? | **Drop down list**  **0=No (GO TO YRB40)**  **1=Yes**  **9 = Prefer not to say (GO TO YRB40)** |
| **YRB35.** Have you deliberately harmed or injured yourself without intending to end your own life during the past 12 months? | **Drop down list**  **0=No (GO TO YRB40)**  **1=Yes**  **9 = Prefer not to say (GO TO YRB40)** |
| **YRB36.** How many times have you ever deliberately harmed or injured yourself without intending to end your own life? | **Drop down list**  **1 = Once**  **2 = Twice**  **3 = Three times**  **4 = More than three times**  **9 = Prefer not to say** |
| **YRB37.** How old were you when you first started to deliberately harm or injure yourself? | **Drop down list**  **1 = 8 years old or younger**  **2 = 9 years old**  **3 = 10 years old**  **4 = 11 years old**  **5 = 12 years old**  **6 = 13 years old**  **7 = 14 years old**  **8 = 15 years old**  **9 = 16 years old**  **10 = 17 years old or older**  **99 = Prefer not to say** |
| **YRB38.**The last time you deliberately hurt or injured yourself without intending to end your own life, what method did you use? **(May have more than one answer. Select all that apply. Scroll down for full list)** | **Drop down list**  **1 = Eating foreign objects**  **2 = Cigarette burns**  **3 = Lighter burns (smilies)**  **4 = Slashing/cutting of skin**  **5 = Biting of skin**  **6 = Attempting to cut off oxygen**  **7 = Banging head against**  **8 = Punching/kicking things repeatedly**  **9 = Stabbing self**  **10 = Scratching or pinching**  **11 = Punching, hitting or slapping**  **12 = Burning or scalding**  **13 = Poisoning or overdosing**  **14= Other**  **99 = Prefer not to say** |
| **YRB39.** During the past 12 months, were you admitted to hospital, treated by a hospital emergency department, or seen by a doctor or nurse as a direct result of injuries caused by an act of deliberate self-harm with no intent to end your own life? **(May have more than one answer. Select all that apply)** | **Drop down list**  **1 = Yes, I was admitted to hospital**  **2 = Yes, I was treated in the hospital emergency department**  **3 = Yes, I was treated by a doctor or nurse**  **4 = No, I did not seek medical help**  **9 = Prefer not to say** |

| SUICIDE (SMHAC 2014) | |
| --- | --- |
| ***Now we are going on to talk about attempted suicide, which is the act of attempting to kill yourself.*** | |
| **YRB40.** Have you ever felt life was not worth living? | **Drop down list**  **0=No**  **1=Yes**  **9= Prefer not to say** |
| **YRB41.** During the past 12 months, did you ever seriously consider attempting suicide? | **Drop down list**  **0=No**  **1=Yes**  **9= Prefer not to say** |
| **YRB42.** During the past 12 months, did you make a plan about how you would attempt suicide? | **Drop down list**  **0=No**  **1=Yes**  **9= Prefer not to say** |
| **YRB43.** Have you ever actually attempted suicide? | **Drop down list**  **0=No (GO TO NEXT MODULE)**  **1=Yes**  **9 – Prefer not to say (GO TO NEXT MODULE)** |
| **YRB44.** Did you attempt suicide in the past 12 months? | **Drop down list**  **0=No**  **1=Yes**  **9= Prefer not to say** |
| **YRB45.** How many times have you ever attempted suicide? | **Drop down list**  **1 = Once**  **2 = Twice**  **3 = Three times**  **4 = More than three times**  **9 = Prefer not to say** |
| **SUIC47. The LAST TIME** attempted suicide, what method did you use? | **Drop down list**  **1 = Eating foreign objects (metal etc)**  **2 = Swallowing poisons**  **3 = Banging head against**  **4 = Punching/kicking things repeatedly**  **5 = Attempted hanging**  **6 = Attempted to cut off oxygen**  **7 = Attempted overdose (alcohol)**  **8 = Attempted overdose (pills)**  **9 = Attempted overdose (heroin)**  **10 = Attempted overdose (other)**  **11 = Attempted overdose (polydrug)**  **12 = Firearms/gunshot**  **13 = Stabbing self**  **14 = Slashing wrists/other body parts**  **15 = Jumping from a height**  **16 = Car accident**  **17 = Other**  **99 = Prefer not to say** |
| **YRB45B.** Which of these statements best describes your situation the last time you attempted suicide? | **Drop down list**  **1 = I made a serious attempt to kill myself and it was only luck that I did not succeed**  **2 = I tried to kill myself, but knew the method may not work**  **3 = My attempt was a cry for help. I did not intend to die**  **9 = Prefer not to say** |
| **YRB46.** During the past 12 months, were you admitted to hospital, treated by a hospital emergency department, or seen by a doctor or nurse as a direct result of injuries caused by an attempt to end your own life? **(May have more than one answer. Select all that apply)** | **Drop down list**  **1 = Yes, I was admitted to hospital**  **2 = Yes, I was treated in the hospital emergency department**  **3 = Yes, I was treated by a doctor or nurse**  **4 = No, I did not seek medical help**  **9 = Prefer not to say** |

| MAJOR DEPRESSION (MINI KID 6.0) | |
| --- | --- |
| **I'm now going to ask you some questions about feeling sad and unhappy.** | |
| **At any time of your life: A1a.** Did you feel sad or depressed? Felt down or empty? Felt grouchy or annoyed? Did you feel this way most of the time, for at least 2 weeks?  **IF 'YES' TO ANY CONTINUE.  IF NO CODE 'NO' TO A1b.** | \| **Drop down list** \| \| --- \| \| **0 = No** \| \| **1 = Yes** \| |
| **A1b.** For the past 2 weeks, did you feel this way, most of the day, nearly every day? | \| **Drop down list** \| \| --- \| \| **0 = No** \| \| **1 = Yes** \| |
| **At any time in your life: A2a.** Were you bored a lot or much less interested in things (Like playing your favorite games)? Have you felt that you couldn't enjoy things? Did you feel this way most of the time, for at least 2 weeks? **IF 'YES' TO ANY CONTINUE.  IF NO CODE 'NO' A2b.** | \| **Drop down list** \| \| --- \| \| **0 = No** \| \| **1 = Yes** \| |
| **A2b.** For the past 2 weeks, did you feel this way, most of the day, nearly every day? | \| **Drop down list** \| \| --- \| \| **0 = No** \| \| **1 = Yes** \| |
|  |  |
| **IS A1a OR A2b CODED YES 🡪 CONTINUE**  **IS A1a OR A2b CODED NO 🡪 GO TO NEXT MODULE**  **IS A1b OR A2b = YES 🡪 EXPLORE CURRENT AND MOST SYMPTOMATIC PAST EPISODE**  **IS A1b AND A2b = NO 🡪 EXPLORE MOST SYMPTOMATIC PAST EPISODE** |  |

|  | **PAST 2 WEEKS** | **PAST EPISODE** |
| --- | --- | --- |
| **A3a.** Were you less hungry or more hungry most days? Did you lose or gain weight without trying? [i.e., by ± 5% of body weight in the past month]? **IF YES TO EITHER, CODE YES** | \| **Drop down list** \| \| --- \| \| **0 = No** \| \| **1 = Yes**  **9 = Prefer not to say** \| | \| **Drop down list** \| \| --- \| \| **0 = No** \| \| **1 = Yes**  **9 = Prefer not to say** \| |
| **A3b.** Did you have trouble sleeping almost every night (“trouble sleeping” means trouble falling asleep, waking up in the middle of the night, waking up too early or sleeping too much)? | \| **Drop down list** \| \| --- \| \| **0 = No** \| \| **1 = Yes**  **9 = Prefer not to say** \| | \| **Drop down list** \| \| --- \| \| **0 = No** \| \| **1 = Yes**  **9 = Prefer not to say** \| |
| **A3c.** Did you talk or move slower than usual? Were you fidgety, restless or couldn’t sit still almost every day? **IF YES TO EITHER, CODE YES** | \| **Drop down list** \| \| --- \| \| **0 = No** \| \| **1 = Yes**  **9 = Prefer not to say** \| | \| **Drop down list** \| \| --- \| \| **0 = No** \| \| **1 = Yes**  **9 = Prefer not to say** \| |
| **A3d.** Did you feel tired most of the time? | \| **Drop down list** \| \| --- \| \| **0 = No** \| \| **1 = Yes**  **9 = Prefer not to say** \| | \| **Drop down list** \| \| --- \| \| **0 = No** \| \| **1 = Yes**  **9 = Prefer not to say** \| |
| **A3e.** Did you feel bad about yourself most of the time? Did you feel guilty most of the time? **IF YES TO EITHER, CODE YES** | \| **Drop down list** \| \| --- \| \| **0 = No** \| \| **1 = Yes**  **9 = Prefer not to say** \| | \| **Drop down list** \| \| --- \| \| **0 = No** \| \| **1 = Yes**  **9 = Prefer not to say** \| |
| **A3ee. IF YES, ASK FOR EXAMPLES.** | *Type in answer* | *Type in answer* |
| **A3f.** Did you have trouble concentrating or did you have trouble making up your mind? **IF YES TO EITHER, CODE YES** | \| **Drop down list** \| \| --- \| \| **0 = No** \| \| **1 = Yes**  **9 = Prefer not to say** \| | \| **Drop down list** \| \| --- \| \| **0 = No** \| \| **1 = Yes**  **9 = Prefer not to say** \| |
| **A3g.** Did you feel so bad that you wished that you were dead? Did you think about hurting yourself? Did you have thoughts of death? Did you think about killing yourself? **IF YES TO ANY, CODE YES** | \| **Drop down list** \| \| --- \| \| **0 = No** \| \| **1 = Yes**  **9 = Prefer not to say** \| | \| **Drop down list** \| \| --- \| \| **0 = No** \| \| **1 = Yes**  **9 = Prefer not to say** \| |
| **A4.** Did these sad, depressed feelings cause a lot of problems at home?  At school? With friends? With other people? Or in some other important way? | \| **Drop down list** \| \| --- \| \| **0 = No** \| \| **1 = Yes**  **9 = Prefer not to say** \| | \| **Drop down list** \| \| --- \| \| **0 = No** \| \| **1 = Yes**  **9 = Prefer not to say** \| |
| **A5.** In between your times of depression, were you free of depression for at least 2 months?  **IF A5 IS CODED ‘YES’, CODE ‘YES’ FOR RECURRENT** | \| **Drop down list** \|  \| \| --- \| --- \| \| **0 = No** \|  \| \| **1 = Yes**  **9 = Prefer not to say** \|  \| | |
| **A6.** How many episodes of depression did you have in your lifetime? Between each episode there must be at least 2 months without any significant depression. | \| **Drop down list**  **0**  **1**  **2**  **3**  **4**  **5**  **6**  **7**  **8**  **9**  **10**  **…**  **20**  **More than 20**  **99=Prefer not to say** \| \| --- \| \|  \| \|  \| | |

| PSYCHOSIS SCREENER (SMHAC 2014) | |
| --- | --- |
| **YCH53.** In the last year, have you ever believed that people were spying on you? | **Drop down list**  **0 = No**  **1 = Yes**  **9 = Prefer not to say** |
| **YCH54.** In the last year, have you ever believed that you were being sent special messages through the TV or radio or that a program had been arranged for you alone and no one else? |  |
| **YCH55.** In the last year, have you ever believed that someone was using special powers to read your mind? |  |
| **YCH56.** In the last year, have you ever seen something or someone that other people who were present could not see, that is had a vision when you were completely awake? |  |
| **YCH57.** In the last year, have you heard things other people could not hear, such as a voice? |  |
| **YCH58.** Did these experiences happen when you were taking drugs or drinking alcohol? | **Drop down list**  **0 = No**  **2 = Yes, I only have these experiences when I have taken drugs or drunk alcohol**  **3 = Yes, but I also have these experiences when I have not taken drugs or drunk alcohol**  **9 = Prefer not to say** |
| **PSY1.** Have you ever been prescribed anti-psychotic medicine or diagnosed with psychosis by a doctor? | **Drop down list**  **0 = No**  **1 = Yes**  **9 = Prefer not to say** |

| ATTENTION DEFICIT HYPERACTIVITY DISORDER (MINI KID 6.0) | |
| --- | --- |
| **Everybody has times when they have trouble concentrating or keeping their mind on what they are doing.** | |
| **ADHD1. Has anyone (teacher, baby sitter, friend or parent) ever complained about your behaviour or performance in school?** | **Drop down list**  **0=No (GO TO NEXT MODULE)**  **1=Yes**  **9 = Prefer not to say** |
| **ADHD2. In the past six months...  a.** Have you often not paid enough attention to details? Made careless mistakes in school? | **Drop down list**  **0=No**  **1=Yes**  **9 = Prefer not to say** |
| **b.** Have you often had trouble keeping your attention focused when playing or doing schoolwork? |  |
| **c.** Have you often been told that you do not listen when others talk directly to you? |  |
| **d.** Have you often had trouble following through with what you were told to do (Like not following through on schoolwork or chores)? Did this happen even though you understood what you were supposed to do? Did this happen even though you weren't trying to be difficult? **IF NO TO ANY, CODE NO** |  |
| **e.** Have you often had a hard time getting organized? |  |
| **f.** Have you often tried to avoid things that make you concentrate or think hard (like schoolwork)? Do you hate or dislike things that make you concentrate or think hard? |  |
| **g.** Have you often lost or forgotten things you needed? Like homework Assignments, pencils, or toys? |  |
| **h.** Do you often get distracted easily by little things (Like sounds or things outside the room)? |  |
| **i.** Do you often forget to do things you need to do every day (Like forget to comb your hair or brush your teeth)? |  |
| **ADHD3. In the past six months:**   **a.** Did you often fidget with your hands or feet? Or did you squirm in your seat? **IF YES TO EITHER, CODE YES** | **Drop down list**  **0=No**  **1=Yes**  **9 = Prefer not to say** |
| **b.** Did you often get out of your seat in class when you were not supposed to? |  |
| **c.** Have you often run around or climbed on things when you weren't supposed to? Did you want to run around or climb on things even though you didn't? **IF YES TO EITHER, CODE YES** |  |
| **d.** Have you often had a hard time playing quietly? |  |
| **e.** Were you always "on the go"? |  |
| **f.** Have you often talked too much? |  |
| **g.** Have you often blurted out answers before the person or teacher has finished the question? |  |
| **h.** Have you often had trouble waiting your turn? |  |
| **i.** Have you often interrupted other people? Like butting in when other people are talking or busy or when they are on the phone? |  |
| **ADHD4. Did you have problems paying attention, being hyper, or impulsive before you were 7 years old?** | **Drop down list**  **0=No**  **1=Yes**  **9 = Prefer not to say** |
| **ADHD5. Did these things cause problems at school? At home? With your family? With your friends?** | **Drop down list**  **0=No**  **1=Yes**  **9 = Prefer not to say** |

| POST-TRAUMATIC STRESS DISORDER (MINI KID 6.0) | |
| --- | --- |
| **Now I’m going to ask you about upsetting things that sometimes happen to children or teenagers.** | |
| **PTSD1**. Has anything really awful ever happened to you? Like being in a flood, tornado or earthquake? Like being in a fire or a really bad accident? Like seeing someone being killed or badly hurt. Have you ever been attacked by someone? | **Drop down list**  **0=No (GO TO NEXT MODULE)**  **1=Yes** |
| **PTSD2.** Did you respond with intense fear, or feel helpless or upset? | **Drop down list**  **0=No (GO TO NEXT MODULE)**  **1=Yes** |
| **PTSD3.** In the past month, has this awful thing come back to you in some way? Like dreaming about it or having a strong memory of it or feeling it in your body? | **Drop down list**  **0=No (GO TO NEXT MODULE)**  **1=Yes** |
| **PTSD4a. In the past month,** have you tried not to think about or talk about this awful thing? **ASK ALL QUESTIONS FROM PTSD4 a to g** | **Drop down list**  **0=No**  **1=Yes** |
| **b.** Have you tried to stay away from things that might remind you of it? |  |
| **c.** Have you had trouble remembering some important part of what happened? |  |
| **d.** Have you been much less interested in your hobbies or your friends? |  |
| **e.** Have you felt cut off from other people? |  |
| **f.** Have you noticed that your feelings are less than before? |  |
| **g.** Have you felt that your life will be shortened or that you will die sooner than other people? |  |
| **SUMMARY OF PTSD4: ARE 3 OR MORE PTSD4 ANSWERS CODED YES?** |  |
| **PTSD5a. In the past month,** have you had trouble sleeping? **ASK ALL QUESTIONS FROM PTSD5 a to e** | **Drop down list**  **0=No**  **1=Yes** |
| **b.** Have you been moody or angry for no reason? |  |
| **c.** Have you had trouble paying attention? |  |
| **d.** Were you nervous or watching out in case something bad might happen? |  |
| **e.** Would you jump when you heard noises? Or when you saw something out of the corner of your eye? I**F YES TO EITHER, CODE YES** |  |
| **SUMMARY OF PTSD5: ARE 2 OR MORE PTSD5 ANSWERS CODED YES?** |  |
| **PTSD6.** In the past month, have these problems upset you a lot? Have they caused you to have problems at school? At home? With your friends? **IF YES TO ANY, CODE YES** | **Drop down list**  **0=No**  **1=Yes** |

| MENTAL HEALTH SERVICE UTILISATION (SMHAC 2014) | |
| --- | --- |
| **The next questions relate to use of services and different types of help that you may have received for emotional and behavioural problems in the last year.** | |
| **SUY5.** In the past 12 months, that is since [MONTH] of last year, have you seen any health professional because of emotional or behavioural problems? **(IF ASKED FOR CLARIFICATION: anxiety, stress, depression, problems concentrating, aggression, hyperactivity)?** | **Drop down list**  **0=No (GO TO SUY4)**  **1=Yes** |
| **SUY6.** Where did you see these health professionals? **(Can have more than one answer, SCROLL TO SEE FULL LIST)** | **Drop down list**  **1 – At school**  **2 – Doctor’s rooms or other private practice**  **3 – Hospital emergency or other outpatient department**  **4 – Child & Adolescent Mental Health Service (CAMHS) or Child & Youth Mental Health Service (CYMHS)**  **5 - Ted Noffs Street University**  **6 - Youth Justice / Juvenile Detention**  **5 - Prison**  **6 – Headspace centre**  **7 – Other public mental health service**  **8 – Other community child or youth health service**  **10 – Other counselling or support service**  **11 – Unsure about where it was** |
|  |  |
| **SUY4.** In the past 12 months have you used a telephone counselling service such as Kids Helpline? | **Drop down list**  **0=No**  **1=Yes** |
| **SUY9.** In the past 12 months have you used Internet-based services to get help or information about emotional or behavioural problems? **This includes searching the Internet or using online services provided by Headspace, Reachout, Youth Beyondblue and others.** | **Drop down list**  **0=No**  **1=Yes** |
| **SUY13.** Have you ever spoken to a counsellor or attended a group program at a drug or alcohol treatment unit or clinic? **If YES,** was in this in the past 12 months? | **Drop down list**  **0 = No**  **1 = Yes, in the past 12 months**  **2 = Yes, but NOT in the past 12 months** |

| REPRODUCTIVE HEALTH (GIRLS ONLY) | |
| --- | --- |
| ***I’m now going to ask you some questions specifically about girls and their health.*** | |
| **MENSTRUATION** |  |
| **REP1.** Have you started your menstrual periods? | **Drop down list**  **0=No (GO TO NEXT MODULE)**  **1=Yes**  **9=Prefer not to say (GO TO REP3)** |
| **REP2**. Do your menstrual periods normally cause you to have heavy periods, pain, discomfort, or any other problems? | **Drop down list**  **0=No**  **1=Yes**  **9 = Prefer not to say** |
| **PREGNANCY** |  |
| **REP3**. Have you ever been pregnant? | **Drop down list**  **0=No (GO TO NEXT MODULE)**  **1=Yes**  **9=Prefer not to say (GO TO REP17)** |
| **REP4.** How old were you when you first became pregnant? | **Drop down list**  **8 = 8 years old or younger**  **9 = 9 years old**  **10 = 10 years old**  **11 = 11 years old**  **12 = 12 years old**  **13 = 13 years old**  **14 = 14 years old**  **15 = 15 years old**  **16 = 16 years old**  **17 = 17 years old**  **99 = Prefer not to say** |
| **REP4a.** For your first pregnancy, was this a planned pregnancy? (i.e. You wanted to get pregnant. It wasn't a surprise.) | **Drop down list**  **0=No**  **1=Yes**  **9 = Prefer not to say** |
| **REP5**. How many times have you been pregnant? | **Drop down list**  **1 = Once**  **2 = Twice**  **3 = Three times**  **4 = Four times**  **5 = Five times**  **6 = More than five times**  **99 = Prefer not to say** |
| **LIVE BIRTHS** |  |
| **REP6.** How many of these pregnancies resulted in you giving birth to a baby? | **Drop down list**  **0 = No live births (GO TO 'REP17')**  **1 = One**  **2 = Two**  **3 = Three**  **4 = Four**  **5 = Five**  **6 = More than five times**  **88 = Still pregnant (GO TO 'REP17')**  **99 = Prefer not to say (GO TO 'REP17')** |
| **REP7.** How old were you when you first gave birth to a baby? | **Drop down list**  **8 = 8 years old or younger**  **9 = 9 years old**  **10 = 10 years old**  **11 = 11 years old**  **12 = 12 years old**  **13 = 13 years old**  **14 = 14 years old**  **15 = 15 years old**  **16 = 16 years old**  **17 = 17 years old**  **99 = Prefer not to say** |
| **REP8.** Do you recall how much your first baby weighed at birth (kg)? **(IF UNSURE, GIVE AN ESTIMATE)** | **Drop down list**  **Less than 1Kg**  **Approx 1 Kg**  **Approx 1.5 Kg**  **Approx 2 kg**  **Approx 2.5 kg**  **Approx 3 kg**  **Approx 3.5 kg**  **Approx 4 kg**  **Approx 4.5 kg**  **Approx 5 kg**  **More than 5 kg**  **888 = Don't know/Unsure**  **999=Prefer not to say** |
| **REP9.** Was your first baby born prematurely (less than 38 weeks)? | **Drop down list**  **0=No**  **1=Yes**  **9=Prefer not to say** |
| **REP10.** Did you have access to prenatal or antenatal care for your first pregnancy? | **Drop down list**  **0=No**  **1=Yes**  **9=Prefer not to say** |
| **REP11.** Did a health provider organise contraception for you immediately after you first gave birth or at the six week visit? | **Drop down list**  **0=No**  **1=Yes**  **9=Prefer not to say** |
| **REP12.** Did you breastfeed your first baby? | **Drop down list**  **0=No**  **1=Yes**  **9=Prefer not to say** |
| **REP13.** Have you ever been clinically diagnosed with post-natal depression? | **Drop down list**  **0=No**  **1=Yes**  **9=Prefer not to say** |
| **REP14.** Thinking about your first child, who have they lived with since they were born? | **Drop down list**  **1=You**  **2=You and your partner**  **3=Your partner**  **4=Their mother and/or father**  **5=Your mother and/or your father**  **6=Other relatives**  **7=Your friends**  **8=Foster family**  **9=Adopted family**  **10=Child welfare institution**  **11=Other**  **88=Don’t know**  **99=Prefer not to say** |
| **REP15**. Who is your first child currently living with? | **Drop down list**  **1=You**  **2=You and your partner**  **3=Your partner**  **4=Their mother and/or father**  **5=Your mother and/or your father**  **6=Other relatives**  **7=Your friends**  **8=Foster family**  **9=Adopted family**  **10=Child welfare institution**  **11=Other**  **88=Don’t know**  **99=Prefer not to say** |
| **REP16.** Have you ever had a child that was given up for adoption? If YES, can you please tell me how many children you have given up for adoption? | **Drop down list**  **0=None given up for adoption**  **1=One**  **2=Two**  **3=Three**  **4=Four**  **5=Five**  **6=More than 5**  **99=Prefer not to say** |
| **OTHER** |  |
| **REP17.** Have you ever had a miscarriage? If yes, how many? | **Drop down list**  **0 - No, never**  **1 – Yes, one**  **2 – Yes, two**  **3 – Yes, three**  **4 – Yes, four**  **5 – Yes, five**  **6 – Yes, more than five times**  **99=Prefer not to say** |
| **REP18.** Have you ever had a stillbirth? If yes, how many? | **Drop down list**  **0 - No, never**  **1 – Yes, one**  **2 – Yes, two**  **3 – Yes, three**  **4 – Yes, four**  **5 – Yes, five**  **6 – Yes, more than five times**  **99=Prefer not to say** |
| **REP19**. Have you ever had a termination of pregnancy (abortion)? If yes, how many? | **Drop down list**  **0 - No, never**  **1 – Yes, one**  **2 – Yes, two**  **3 – Yes, three**  **4 – Yes, four**  **5 – Yes, five**  **6 – Yes, more than five times**  **99=Prefer not to say** |
| **REP20.** Have you ever smoked cigarettes during any of your pregnancies? | **Drop down list**  **0=No**  **1=Yes**  **9=Prefer not to say** |
| **REP21.** Have you ever drunk any alcohol during any of your pregnancies? | **Drop down list**  **0=No**  **1=Yes**  **9=Prefer not to say** |
| **REP21a.** While you were pregnant, on how many days have you had at least one drink of alcohol? | \| **Drop down list** \| \| --- \| \| **1** \| \| **2** \| \| **3** \| \| **4** \| \| **5** \| \| **6** \| \| **7** \| \| **8** \| \| **9** \| \| **10** \| \| **11** \| \| **12** \| \| **13** \| \| **14** \| \| **15** \| \| **16** \| \| **17** \| \| **18** \| \| **19** \| \| **20** \| \| **21** \| \| **22** \| \| **23** \| \| **24** \| \| **25** \| \| **26** \| \| **27** \| \| **28** \| \| **29** \| \| **30** \| \| **More than 30 days** \| \| **99 = Prefer not to say** \| |
| **REP21b.** On the days that you drank alcohol during your pregnancy/ies, on average how many standard drinks did you drink per day?  **(A standard drink is a small glass of wine or middy/pot/half pint of beer, a nip of spirits or a mixed drink)** | \| **Drop down list** \| \| --- \| \| **1** \| \| **2** \| \| **3** \| \| **4** \| \| **5** \| \| **6** \| \| **7** \| \| **8** \| \| **9** \| \| **10** \| \| **11** \| \| **12** \| \| **13** \| \| **14** \| \| **15** \| \| **16** \| \| **17** \| \| **18** \| \| **19** \| \| **20** \| \| **21** \| \| **22** \| \| **23** \| \| **24** \| \| **25** \| \| **26** \| \| **27** \| \| **28** \| \| **29** \| \| **30** \| \| **More than 30** \| \| **99 = Prefer not to say** \| |
| **REP22.** Have you ever taken any illicit drugs during any of your pregnancies? | **Drop down list**  **0=No**  **1=Yes**  **9=Prefer not to say** |

| FATHERS AND CHILDREN (BOYS ONLY) | |
| --- | --- |
| **PAR1.** Have you ever made a girl or woman pregnant? | **Drop down list**  **0=No (GO TO NEXT MODULE)**  **1=Yes**  **8=Unsure/don't know (GO TO NEXT MODULE)**  **9=Prefer not to say (GO TO NEXT MODULE)** |
| **a.** **IF YES,** how many times? | **Drop down list**  **1 = Once only**  **2 = Two**  **3 = Three**  **4 = Four**  **5 = Five**  **6 = More than five times**  **9 = Prefer not to say** |
| **PAR2.** Do you have any children? | **Drop down list**  **0=No (GO TO NEXT MODULE)**  **1=Yes**  **8=Unsure/don't know (GO TO NEXT MODULE)**  **9=Prefer not to say (GO TO NEXT MODULE)** |
| **a.** **If YES,** how many children? | **Drop down list**  **1 = One**  **2 = Two**  **3 = Three**  **4 = Four**  **5 = Five**  **6 = More than five**  **9 = Prefer not to say** |
| **b.** How old were you when your first child was born? | **Drop down list**  **8 = 8 years old or younger**  **9 = 9 years old**  **10 = 10 years old**  **11 = 11 years old**  **12 = 12 years old**  **13 = 13 years old**  **14 = 14 years old**  **15 = 15 years old**  **16 = 16 years old**  **17 = 17 years old**  **99 = Prefer not to say** |
| **c.** Thinking about your first child, who have they lived with since they were born? | **Drop down list**  **1 = You**  **2 = You and your partner**  **3 = Your partner**  **4 = Their mother and/or father**  **5 = Your mother and/or your father**  **6 = Other relatives**  **7 = Your friends**  **8 = Foster family**  **9 = Adopted family**  **10 = Child welfare institution**  **11 = Other**  **88 = Don’t know**  **99 = Prefer not to say** |
| **PAR3.** Have you ever had a child that was given up for adoption? If yes, can you please tell me how many children you have been given up for adoption? | **Drop down list**  **0 = None given up for adoption**  **1 = One**  **2 = Two**  **3 = Three**  **4 = Four**  **5 = Five**  **6 = More than 5**  **9 = Prefer not to say** |

| SEXUAL IDENTITY, ATTRACTION AND SEXUAL HISTORY | |
| --- | --- |
| **Now, thinking about yourself and your sexual identity and history…** | |
| **SEXID1.** Do you think of yourself as heterosexual or straight, homosexual (gay)/lesbian, or bisexual? | **Drop down list**  **1 = Heterosexual or straight**  **2 = Homosexual or lesbian**  **3 = Bisexual**  **4 = Queer**  **5 = Not sure/Undecided**  **6 = Something else/other**  **99 = Don’t want to say** |
| **SEXID2.** Which of these statements best describes your sexual feelings at the moment? **(READ OUT DROP DOWN LIST 1-3)** | **Drop down list**  **1 = I am attracted only to people of the opposite sex**  **2 = I am attracted to people of both sexes**  **3 = I am attracted only to people of my own sex**  **4 = Not sure**  **99 = Prefer not to say** |
| **SEX1.** Can you tell me if you have ever had sex? By this I mean vaginal, anal or oral sex. This does not include masturbation. | **Drop down list**  **0=Never had sex (GO TO SEX19)**  **1=Yes**  **99=Prefer not to say (GO TO SEX19)** |
| **SEX2.** Ideally, how often would you like to have sex? | **Drop down list**  **0 = Don't want to have sex**  **1 = Every day**  **2 = Once a week**  **3 = Twice a week**  **4 = 3 times a week**  **5 = 4 times a week**  **6 = 5 times a week**  **7 = 6 times a week**  **8 = Once every fortnight**  **9 = Once a month**  **10 = Once every 2 months**  **11 = Once every 3 months**  **11 = Once every 6 months**  **12 = Once a year**  **99 = Prefer not to say** |
| **SEX3.** During your life, with how many people have you had sex? **(IF UNSURE, ASK FOR AN ESTIMATE)** | \| **Drop down list** \| \| --- \| \| **1** \| \| **2** \| \| **3** \| \| **4** \| \| **5** \| \| **6** \| \| **7** \| \| **8** \| \| **9** \| \| **10** \| \| **….** \| \| **99** \| \| **100 or more** \| \| **999=Prefer not to say** \| |
| **a.** And how often did you use a condom with them? **Is that all the time, most of the time, half the time, some of the time or never?** | **Drop down list**  **1=All the time**  **2=Most of the time**  **3=Half the time**  **4=Some of the time**  **5=Never** |
| **SEX4.** Can you tell me how old you were when you first had sex? By this I mean vaginal, anal or oral sex. This does not include masturbation. | **Drop down list**  **1 - 1 year old or less**  **2 - 2 years old**  **3 - 3 years old**  **4 - 4 years old**  **5 - 5 years old**  **6 - 6 years old**  **7 - 7 years old**  **8 - 8 years old**  **9 - 9 years old**  **10 - 10 years old**  **11 - 11 years old**  **12 - 12 years old**  **13 - 13 years old**  **14 - 14 years old**  **15 - 15 years old**  **16 - 16 years old**  **17 - 17 years old**  **99 = Prefer not to say** |
| **a.** Was your first sexual partner male, female or other? | **Drop down list**  **1=Male**  **2=Female**  **3=Transgender**  **99=Prefer not to say** |
| **b.** How old was he or she? **(IF UNSURE, ASK APPROXIMATE AGE)** | **Drop down list**  **1 year old or less**  **2 years old**  **3 years old**  **4 years old**  **5 years old**  **6 years old**  **7 years old**  **8 years old**  **9 years old**  **10 years old**  **11 years old**  **12 years old**  **13 years old**  **14 years old**  **15 years old**  **16 years old**  **17 years old**  **18 years old**  **...**  **>65 years old**  **999=Prefer not to say** |
| **SEX5.** Have you ever had oral sex? | **Drop down list**  **0=No (GO TO SEX6)**  **1=Yes**  **99=Prefer not to say (GO TO SEX6)** |
| **a.** How old were you when you first gave or received oral sex? | **Drop down list**  **1 - 1 year old or less**  **2 - 2 years old**  **3 - 3 years old**  **4 - 4 years old**  **5 - 5 years old**  **6 - 6 years old**  **7 - 7 years old**  **8 - 8 years old**  **9 - 9 years old**  **10 - 10 years old**  **11 - 11 years old**  **12 - 12 years old**  **13 - 13 years old**  **14 - 14 years old**  **15 - 15 years old**  **16 - 16 years old**  **17 - 17 years old**  **99 = Prefer not to say** |
| **SEX6.** Have you ever had vaginal sex? | **Drop down list**  **0=No (GO TO SEX7)**  **1=Yes**  **99=Prefer not to say (GO TO SEX7)** |
| **a.** How old were you when you first had vaginal sex? | **Drop down list**  **1 - 1 year old or less**  **2 - 2 years old**  **3 - 3 years old**  **4 - 4 years old**  **5 - 5 years old**  **6 - 6 years old**  **7 - 7 years old**  **8 - 8 years old**  **9 - 9 years old**  **10 - 10 years old**  **11 - 11 years old**  **12 - 12 years old**  **13 - 13 years old**  **14 - 14 years old**  **15 - 15 years old**  **16 - 16 years old**  **17 - 17 years old**  **99 = Prefer not to say** |
| **b.** And how often did you use a condom when you had vaginal sex? **Is that all the time, most of the time, half the time, some of the time or never?** | **Drop down list**  **1=All the time**  **2=Most of the time**  **3=Half the time**  **4=Some of the time**  **5=Never** |
| **SEX7.** Have you ever had anal sex? | **Drop down list**  **0=No (GO TO SEX8)**  **1=Yes**  **99=Prefer not to say (GO TO SEX8)** |
| **a.** How old were you when you first had anal sex? | **Drop down list**  **1 - 1 year old or less**  **2 - 2 years old**  **3 - 3 years old**  **4 - 4 years old**  **5 - 5 years old**  **6 - 6 years old**  **7 - 7 years old**  **8 - 8 years old**  **9 - 9 years old**  **10 - 10 years old**  **11 - 11 years old**  **12 - 12 years old**  **13 - 13 years old**  **14 - 14 years old**  **15 - 15 years old**  **16 - 16 years old**  **17 - 17 years old**  **99 = Prefer not to say** |
| **b.** And how often did you use a condom when you had anal sex? **Is that all the time, most of the time, half the time, some of the time or never?** | **Drop down list**  **1=All the time**  **2=Most of the time**  **3=Half the time**  **4=Some of the time**  **5=Never** |
| **SEX9.** In the last 12 months, how many people have you had sex with? This includes vaginal, anal or oral sex and not masturbation. | \| **Drop down list** \| \| --- \| \| **0 (GO TO SEX10)**  **1** \| \| **2** \| \| **3** \| \| **4** \| \| **5** \| \| **6** \| \| **7** \| \| **8** \| \| **9** \| \| **10** \| \| **….** \| \| **99** \| \| **100 or more** \| \| **999=Prefer not to say (GO TO SEX10)** \| |
| **a.** If yes, did you have sex with males only, females only or both? | **Drop down list**  **1 = Males only**  **2 = Females only**  **3 = Both males and females**  **9 = Prefer not to say** |
| **b.** And how often did you use a condom? **Is that all the time, most of the time, half the time, some of the time or never?** | **Drop down list**  **1=All the time**  **2=Most of the time**  **3=Half the time**  **4=Some of the time**  **5=Never** |
| **SEX10.** Thinking back to the LAST TIME you had sex. Was the last person you had sex with male, female or other? | **Drop down list**  **1=Male**  **2=Female**  **3=Transgender**  **99=Prefer not to say** |
| **a.** Was the last person you had sex with ...? **(READ OUT DROP DOWN LIST 1-4)** | **Drop down list**  **1=Someone you had just met for the first time?**  **2=Someone you had known for a while, but had not had sex with before?**  **"3=Someone known for a while, had sex with before,**  **but not current gf/bf?"**  **4=Your current girlfriend (gf)/boyfriend (bf)?**  **99=Prefer not to say** |
| **b.** How old was the last person you had sex with? **(IF UNSURE, ASK APPROXIMATE AGE)** | **Drop down list**  **1 - 1 year old or less**  **2 - 2 years old**  **3 - 3 years old**  **4 - 4 years old**  **5 - 5 years old**  **6 - 6 years old**  **7 - 7 years old**  **8 - 8 years old**  **9 - 9 years old**  **10 - 10 years old**  **11 - 11 years old**  **12 - 12 years old**  **13 - 13 years old**  **14 - 14 years old**  **15 - 15 years old**  **16 - 16 years old**  **17 - 17 years old**  **99 = Prefer not to say** |
| **c.** And did you use a condom with the last person you had sex with? | **Drop down list**  **0=No**  **1=Yes**  **99=Prefer not to say** |
| **SEX11.** Thinking back to the last time you had sex, BEFORE you had sex, did you talk to this person about… **(READ OUT)** |  |
| **a.** Avoiding pregnancy? | **Drop down list**  **0=No**  **1=Yes**  **99=Prefer not to say** |
| **b.** Avoiding HIV infection? | **Drop down list**  **0=No**  **1=Yes**  **99=Prefer not to say** |
| **c.** Avoiding other sexually transmissible infections? | **Drop down list**  **0=No**  **1=Yes**  **99=Prefer not to say** |
| **d.** Using a condom? | **Drop down list**  **0=No**  **1=Yes**  **99=Prefer not to say** |
| **SEX12.** The last time you had sex, did you or your partner use anything to prevent pregnancy or sexually transmitted infections (STIs)? | **Drop down list**  **0=No**  **1=Yes**  **99=Prefer not to say** |
| **If YES,** what method did you use? **(DO NOT READ OUT)** |  |
| **a.** Birth control pills and condoms | **Drop down list**  **1=Yes** |
| **b.** Birth control pills | **Drop down list**  **1=Yes** |
| **c.** Condoms | **Drop down list**  **1=Yes** |
| **d.** Withdrawal | **Drop down list**  **1=Yes** |
| **e.** Some other method | **Type in other method** |
| **f.** Not sure | **Drop down list**  **1=Yes** |
| **SEX13.** Were you drunk or high last time you had sex? | **Drop down list**  **0=No**  **1=Yes**  **99=Prefer not to say** |
| **SEX14.** The last time you had sex, did you want to have sex? | **Drop down list**  **0=No**  **1=Yes**  **99=Prefer not to say** |
| **Thinking about other times you had sex…** |  |
| **SEX15.** Have you ever had sex to get drugs or money? | **Drop down list**  **0=No (GO TO SEX16)**  **1=Yes**  **99=Prefer not to say (GO TO SEX16)** |
| **a.** In your lifetime, how many times has this happened? **(IF UNSURE, ASK FOR AN ESTIMATE)** | \| **Drop down list** \| \| --- \| \| **1** \| \| **2** \| \| **3** \| \| **4** \| \| **5** \| \| **6** \| \| **7** \| \| **8** \| \| **9** \| \| **10** \| \| **….** \| \| **99** \| \| **100 or more times** \| \| **999=Prefer not to say** \| |
| **b.** And how often did you use a condom? **Is that all the time, most of the time, half the time, some of the time or never?** | **Drop down list**  **1=All the time**  **2=Most of the time**  **3=Half the time**  **4=Some of the time**  **5=Never** |
| **SEX16. Have you ever worked as a sex worker?** | **Drop down list**  **0=No (GO TO SEX17)**  **1=Yes**  **99=Prefer not to say (GO TO SEX17)** |
| **a.** In that time, how often did you use a condom? **Is that all the time, most of the time, half the time, some of the time or never?** | **Drop down list**  **1=All the time**  **2=Most of the time**  **3=Half the time**  **4=Some of the time**  **5=Never** |
| **SEX17.** In your lifetime, have you ever had sex when you didn’t want to?  **If YES,** was it because…. **(READ OUT)** | **Drop down list**  **0=No (GO TO SEX18)**  **1=Yes**  **99=Prefer not to say (GO TO SEX18)** |
| **a.** You were too drunk at the time? | **Drop down list**  **0=No**  **1=Yes**  **99=Prefer not to say** |
| **b.** You were too high at the time? | **Drop down list**  **0=No**  **1=Yes**  **99=Prefer not to say** |
| **c.** Because your partner thought you should? | **Drop down list**  **0=No**  **1=Yes**  **99=Prefer not to say** |
| **d.** Because your friends thought you should? | **Drop down list**  **0=No**  **1=Yes**  **99=Prefer not to say** |
| **e.** Because you were forced or frightened at the time? | **Drop down list**  **0=No**  **1=Yes**  **99=Prefer not to say** |
| **f.** Other reason | **Drop down list**  **0=No**  **1=Yes**  **99=Prefer not to say** |
| **Now, thinking about your other experiences:** |  |
| **SEX19.** In your lifetime did someone ever try to: |  |
| **a.** Physically hurt you (eg: hit, slap or kick you) | **Drop down list**  **0=No**  **1=Yes**  **99=Prefer not to say** |
| **b.** Tried to limit your contact with family or friends? | **Drop down list**  **0=No**  **1=Yes**  **99=Prefer not to say** |
| **c.** Verbally abused you (called you names to put you down or make you feel bad? | **Drop down list**  **0=No**  **1=Yes**  **99=Prefer not to say** |
| **d.** Stopped you knowing about or having access to money? | **Drop down list**  **0=No**  **1=Yes**  **99=Prefer not to say** |
| **The next question(s) are about the time you use the internet which is not related to your school work or for work purposes.** |  |
| **INT1.** Do you use the internet on a computer, laptop, smartphone, mobile phone, tablet or other hand held device? Internet use includes accessing social media such as Facebook or Twitter, emailing, looking at websites or chatting online. | **Drop down list**  **0=No (GO TO NEXT MODULE)**  **1=Yes**  **99=Prefer not to say (GO TO NEXT MODULE)** |
| **SEX20.** Do you use any social networking sites?  **If YES**, can you tell me which social networking sites do you use regularly? **(DO NOT READ OUT)** | **Drop down list**  **0=No**  **1=Yes**  **99=Prefer not to say** |
| **a.** Facebook | **Drop down list**  **1=Yes** |
| **b.** Twitter |  |
| **c.** Snapchat |  |
| **d.** Tumblr |  |
| **e.** YouTube |  |
| **f.** MySpace |  |
| **g.** reddit |  |
| **h.** Instagram |  |
| **i.** Flickr |  |
| **j.** GooglePlus |  |
| ***l.*** *Other* | ***Type in answers*** |
| ***m.*** *Other* | ***Type in answers*** |
| ***n.*** *Other* | ***Type in answers*** |
| **SEX21.** Have you ever used the Internet or social networking websites to look for potential sexual partners? | **Drop down list**  **0=No (GO TO SEX23a)**  **1=Yes**  **99=Prefer not to say (GO TO SEX 23a)** |
| **SEX22.** Have you met a potential sexual partner in person (face-to-face) that you met first on an Internet website or social networking website? **[If asked to clarify e.g. Facebook, Tinder, Plenty of Fish etc.]** | **Drop down list**  **0=No (GO TO SEX23a)**  **1=Yes**  **99=Prefer not to say (GO TO SEX 23a)** |
| **a.** Which Internet site or social networking site did you use to find this partner? | ***Type in answers*** |
| **b.** How many did you meet in person? **(IF UNSURE, ASK FOR AN ESTIMATE)** | \| **Drop down list** \| \| --- \| \| **1** \| \| **2** \| \| **3** \| \| **4** \| \| **5** \| \| **6** \| \| **7** \| \| **8** \| \| **9** \| \| **10** \| \| **….** \| \| **99** \| \| **100 or more** \| \| **999=Prefer not to say** \| |
| **c.** Of these people, how many have you had sex with? This includes vaginal or anal sex and does not include masturbation**. (IF UNSURE, ASK FOR AN ESTIMATE)** | \| **Drop down list** \| \| --- \| \| **1** \| \| **2** \| \| **3** \| \| **4** \| \| **5** \| \| **6** \| \| **7** \| \| **8** \| \| **9** \| \| **10** \| \| **….** \| \| **99** \| \| **100 or more** \| \| **999=Prefer not to say** \| |
| **d.** And how often did you use a condom? **Is that all the time, most of the time, half the time, some of the time or never?** | **Drop down list**  **1=All the time**  **2=Most of the time**  **3=Half the time**  **4=Some of the time**  **5=Never** |
| **The next questions are about what people might do on their computers, mobile phones or other handheld devices.** | **Drop down list**  **0=No**  **1=Yes**  **99=Prefer not to say** |
| **SEX23a.** Have you ever sent a sexually explicit written text message to someone |  |
| **SEX23b.** Have you ever have received a sexually explicit written text message |  |
| **SEX23c.** Have you ever sent someone a sexually explicit nude or nearly nude photo or video of yourself |  |
| **SEX23d.** Have you ever received a sexually explicit nude or nearly nude photo or video of someone else |  |
| **SEX24a.** Has someone ever shared private and sexually explicit nude or nearly nude photos or videos of yourself without your consent |  |
| **SEX24b.** Have you ever shared private and sexually explicit nude or nearly nude photos or videos of someone else without their consent |  |

| PERCEPTIONS OF RISK | |
| --- | --- |
| **Now I would like to talk to you about your chances of getting different types of infections. Please answer if you are LIKELY or NOT LIKELY to get a particular infection.** | |
| **RISK1.** How likely do you think you are personally to get HIV infection? | **Drop down list**  **1 = Likely**  **2 = Not likely**  **8 = Never heard/Don't know what that is (GO TO RISK2)** |
| **a.** Why is that? (Type in answer) | *Type in answer* |
| **RISK2.** How likely do you think you are personally to get any sexually transmitted infections or STIs? | **Drop down list**  **1 = Likely**  **2 = Not likely**  **8 = Never heard/Don't know what that is (GO TO RISK3)** |
| **a.** Why is that? (Type in answer) | *Type in answer* |
| **RISK3.** How likely do you think you are personally to get hepatitis C? | **Drop down list**  **1 = Likely**  **2 = Not likely**  **8 = Never heard/Don't know what that is (GO TO RISK4)** |
| **a.** Why is that? (Type in answer) | *Type in answer* |
| **RISK4.** How likely do you think you are personally to get HPV or the human papilloma virus? | **Drop down list**  **1 = Likely**  **2 = Not likely**  **8 = Never heard/Don't know what that is (GO TO NEXT MODULE)** |
| **a.** Why is that? (Type in answer) | *Type in answer* |

| KNOWLEDGE OF HIV, STIs, HEPATITIS | |
| --- | --- |
| **We are now going to ask you some statements which you need to answer either TRUE OR FALSE.** | |
| **KNOW1.** A man can have a sexually transmissible infection without any obvious symptoms. | **Drop down list**  **1=True**  **2=False**  **8=Don't know**  **9=Prefer not to say** |
| **KNOW2.** People who always use condoms are safe from all STIs. |  |
| **KNOW3.** A woman can have a sexually transmissible infection without any obvious symptoms |  |
| **KNOW4.** Chlamydia is easily treated with antibiotics. |  |
| **KNOW5**. Apart from HIV, all sexually transmissible infections can be cured. |  |
| **KNOW6.** Chlamydia is a sexually transmissible infection that affects only women. |  |
| **KNOW7.** Chlamydia can lead to infertility in women |  |
| **KNOW8.** Once a person has caught genital herpes, then they will always have the virus |  |
| **KNOW9.** Gonorrhoea can be transmitted during oral sex. |  |
| **KNOW10.** Genital warts can only be spread by intercourse. |  |
| **KNOW11.** HIV only infects gay men and injecting drug users. |  |
| **KNOW12.** Cold sores and genital herpes can be caused by the same virus. |  |

| SEXUAL HEALTH EDUCATION AND INFORMATION | |
| --- | --- |
| **The next questions are about where you go to get advice and information about your sexual health.** |  |
| **SEXED1.** Have you ever sought information or advice about sexually transmitted infections or diseases (including HIV/AIDS)?  **If YES,** who did you speak to or where did you go to for information or advice? **(DO NOT READ OUT)** | **Drop down list**  **0=No**  **1=Yes**  **9=Prefer not to say** |
| **a.**        Aboriginal Medical Service / Aboriginal Health Service | **Drop down list**  **1=Yes** |
| **b.**        Local doctor at a General Practice clinic |  |
| **c.**        Family planning clinic / sexual health clinic /community health service |  |
| **d.**        Teacher |  |
| **e.**        Boyfriend/ girlfriend |  |
| **f.**         Female friend |  |
| **g.**        Male friend |  |
| **h.**        Parent |  |
| **i.**         Older brother |  |
| **j.**         Older sister |  |
| **k.**        Relatives |  |
| **l.**         Youth worker |  |
| **m.**      Internet |  |
| **n.**        Magazines |  |
| **o.**        Pamphlets |  |
| **p.**        Media |  |
| **q.**        Other | *Type in answers* |
| **SEXED2.** In the past 12 months have you used the Internet or online services to get help or information about your sexual health including, sexually transmitted infections or HIV/AIDS?  **IF YES,** what internet-based services did you access...? **(READ OUT LIST BELOW, Select all that apply)** | **Drop down list**  **0=No**  **1=Yes**  **9=Prefer not to say** |
| **a.** Internet websites | **Drop down list**  **0=No**  **1=Yes**  **9=Prefer not to say** |
| **b.** Social networking sites e.g. Facebook |  |
| **c.** Chat room or support group |  |
| **d.** Online personal support or counselling |  |
| **e.** Online self-help |  |
| **f.** Online information about sexual health services in the community |  |
| **SEXED3.** Have you ever accessed Aboriginal Health Services in the community? | **Drop down list**  **0=No**  **1=Yes**  **9=Prefer not to say** |

| HPV VACCINATIONS | |
| --- | --- |
| **READ OUT TO GIRLS ONLY: From 2007, a HPV vaccine (brand name Gardasil) that can prevent cervical cancer was provided free to all girls and women aged 12 to 26 years of age in Australia.** [HPV means Human Papilloma Virus] | |
| **READ OUT TO BOYS ONLY: From 2013, a HPV vaccine (brand name Gardasil) that can prevent cancers of the anus, mouth/throat and penis was provided for free to all adolescent boys aged 12 years and over in Australia.**  [HPV means Human Papilloma Virus] | |
| **HPV1.** Have you ever been vaccinated against HPV? **[IF VACCINATED OVERSEAS CODE AS 1. YES]** | **Drop down list**  **0=No**  **1=Yes (GO TO NEXT QUESTION HPV2)**  **7=Never heard of the HPV vaccine**  **8=Don't know/Unsure**  **9=Prefer not to say** |
| **HPV1a.** Do you want the HPV vaccine to protect you against **[GIRLS: cervical cancer] [BOYS: cancers of the anus, mouth/throat, or penis]?** | **Drop down list**  **0=No (GO TO NEXT MODULE)**  **1=Yes (GO TO NEXT MODULE)**  **8=Don't know/Unsure (GO TO NEXT MODULE)**  **9=Prefer not to say (GO TO NEXT MODULE)** |
| **HPV2.** How many doses of HPV vaccine did you receive? | **Drop down list**  **0 = None**  **1 = 1 dose**  **2 = 2 doses**  **3 = 3 doses**  **4 = Don’t know/Unsure**  **9 = Prefer not to say** |

| STI, HIV, HCV TESTING AND DIAGNOSIS | |
| --- | --- |
| **The next few questions are about sexually transmitted infections.** | |
| **STI1.** Have you ever been tested for a STI (sexually transmitted infection)? Have you ever been tested for HIV/AIDS (human immunodeficiency virus)?  **ANY YES, CODE 'YES'.  NO TO ALL THEN CODE 'NO'** | **Drop down list**  **0=Never tested (GO TO NEXT MODULE)**  **1 = Yes, in the last year**  **2 = Yes, more than a year ago**  **8=Don't know/Unsure (GO TO NEXT MODULE)**  **9=Prefer not to say (GO TO NEXT MODULE)** |
| **STI2.** Where did you get your last STI and/or HIV test? | **Drop down list**  **1 = At an Aboriginal Medical Service**  **2 = Local doctor at a General Practice clinic**  **3 = Family planning clinic / Sexual health clinic**  **4 = Youth Justice/Juvenile Justice/Prison**  **5 = Hospital**  **6 = No treatment**  **7 = Other**  **9 = Prefer not to say** |
| **STI3.** Have you ever been diagnosed with a sexually transmissible infection (STI) and/or HIV/AIDS? | **Drop down list**  **0=No (GO TO NEXT MODULE)**  **1 = Yes, in the last year**  **2 = Yes, more than a year ago**  **9=Prefer not to say (GO TO NEXT MODULE)** |
| **STI4.** Which infections have you been diagnosed with? **(DO NOT READ OUT)** |  |
| **a.** Chlamydia | **Drop down list**  **1=Yes** |
| **b.** Gonorrhea |  |
| **c.** Syphilis |  |
| **d.** Trichomoniasis (Trich) |  |
| **e.** Herpes |  |
| **f.** HPV (Genital Warts) |  |
| **g.** HIV/AIDS |  |
| **h.** Other | ***Type in answer*** |
| **STI5.** Where did you go for treatment? | **Drop down list**  **1 = At an Aboriginal Medical Service**  **2 = Local doctor at a General Practice clinic**  **3 = Family planning clinic / Sexual health clinic**  **4 = Youth Justice/Juvenile Justice/Prison**  **5 = Hospital**  **6 = No treatment**  **7 = Other**  **9 = Prefer not to say** |

| ALCOHOL DEPENDENCE/ABUSE (SMHAC2014 & MINI KID 6.0) | |
| --- | --- |
| **I am now going to ask you about alcohol and drinking** | |
| **YRB8.** Have you ever had a drink of alcohol, other than a few sips? | **Drop down list**  **0=No, never drank or just a few sips**  **1=Yes, drank alcohol**  **9=Prefer not to say** |
| **YRB9.** How old were you when you first did this? | **Drop down list**  **1 year old or less**  **2 years old**  **3 years old**  **4 years old**  **5 years old**  **6 years old**  **7 years old**  **8 years old**  **9 years old**  **10 years old**  **11 years old**  **12 years old**  **13 years old**  **14 years old**  **15 years old**  **16 years old**  **17 years old**  **(99 = Don't want to say)** |
| **YRB10.** Since you were **[AGE FROM YRB9]**, on how many days have you had at least one drink of alcohol? | **Drop down list**  **1 or 2 days**  **3 to 4 days**  **5 to 9 days**  **10 to 19 days**  **20 or more days**  **999 = Prefer not to say** |
| **ALC1a.** In the past year, have you had 3 or more drinks of alcohol in a day? | **Drop down list**  **0=No (GO TO YRB11)**  **1=Yes** |
| **ALC1b.** At those times, did you have 3 or more drinks in 3 hours? | **Drop down list**  **0=No (GO TO YRB11)**  **1=Yes** |
| **ALC1c.** Did you do this 3 or more times in the past year? | **Drop down list**  **0=No (GO TO YRB11)**  **1=Yes** |
| **ALC2. In the past year:** |  |
| **a.** Did you need to drink a lot more alcohol to get the same feeling you got when you first started drinking? | **Drop down list**  **0 = No**  **1 = Yes**  **9 = Prefer not to say** |
| **b.** In the past year, whenever you cut down on drinking or stopped drinking, did your hands shake? Did you sweat? Did you feel nervous or like you couldn't sit still? Did you ever drink to keep from getting those problems? Did you drink again to keep from getting a hangover?  **IF YES TO ANY, CODE YES** | **Drop down list**  **0 = No**  **1 = Yes**  **9 = Prefer not to say** |
| **c.** In the past year, when you drank alcohol, did you end up drinking more than you had planned to? | **Drop down list**  **0 = No**  **1 = Yes**  **9 = Prefer not to say** |
| **d.** In the past year, have you tried to cut down or stop drinking alcohol but were not able to? | **Drop down list**  **0 = No**  **1 = Yes**  **9 = Prefer not to say** |
| **e.** In the past year, on days when you drank, did you spend more than three hours doing it? Count the time it took you to get the alcohol, drink it, and get over it. | **Drop down list**  **0 = No**  **1 = Yes**  **9 = Prefer not to say** |
| **f.** In the past year, did you spend less time on other things because of your drinking (Like school, hobbies, or being with friends)? | **Drop down list**  **0 = No**  **1 = Yes**  **9 = Prefer not to say** |
| **g.** In the past year, did your drinking cause problems with your health or your mind? Did you keep on drinking even though you knew that it caused these problems? | **Drop down list**  **0 = No**  **1 = Yes**  **9 = Prefer not to say** |
| **ALC3. In the past year:**  **a.** Were you drunk or hung-over more than once when you had something important to do, like schoolwork or responsibilities at home? Did this cause any problems?  **CODE YES ONLY IF THIS CAUSED PROBLEMS** | **Drop down list**  **0 = No**  **1 = Yes**  **9 = Prefer not to say** |
| **b.** In the past year, were you drunk more than once while doing something risky (Like riding a bike, driving a car or boat, or using machines)? | **Drop down list**  **0 = No**  **1 = Yes**  **9 = Prefer not to say** |
| **c.** In the past year, did you have legal problems more than once because of your drinking (Like getting arrested or stopped by the police)? | **Drop down list**  **0 = No**  **1 = Yes**  **9 = Prefer not to say** |
| **d.** In the past year, did you keep drinking even if your drinking caused problems with your family or with other people? | **Drop down list**  **0 = No**  **1 = Yes**  **9 = Prefer not to say** |
| **Thinking back over the last 30 days…** |  |
| **YRB11.** Have you had a drink of alcohol, other than a few sips in the last 30 days? | **Drop down list**  **0 = No (GO TO NEXT MODULE)**  **1 = Yes**  **9 = Prefer not to say** |
| **YRB12.** During the past 30 days, on the days that you drank alcohol on average how many standard drinks did you drink per day? **(A standard drink is a small glass of wine or middy/pot/half pint of beer, a nip of spirits or a mixed drink)** | **Drop down list**  **1 = One drink**  **2 = Two drinks**  **3 = Three drinks**  **4 = Four drinks**  **5 = 5 or more drinks**  **9 = Prefer not to say** |
| **YRB13.** During the last 30 days, on how many days did you have 4 or more standard drinks of alcohol in a row, that is, within a couple of hours? | \| **Drop down list** \| \| \| --- \| --- \| \| **0 = 0 days** \| \| \| **1 = One day** \| \| \| **2 = Two days** \| \| \| **3 = Three to four days** \| \| \| **4 = Five to nine days** \| \| \| **6 = Ten to 19 days** \| \| \| **7 = 20 or more days** \| \| \| **9 = Prefer not to say** \| \| |
| **YRB14.** During the last 30 days, what is the largest number of standard alcoholic drinks you had in a row, that is, within a couple of hours? | **Drop down list**  **1 or 2 drinks**  **3 drinks**  **4 drinks**  **5 drinks**  **6 or 7 drinks**  **8 or 9 drinks**  **10 or more**  **9 - Prefer not to say** |
| **YRB15.** What is the main reason that you decided to drink alcohol? | **Drop down list**  **1 - Because you like the feeling, you want to get high or have fun**  **2 - Because it helps you enjoy parties or social gatherings more**  **3 - Because you want to fit in, you want people to like you or you don’t want to feel left out**  **4 - Because it helps cheer you up when you are in a bad mood**  **5 - Because it helps cheer you up when you feel depressed or nervous**  **6 - Because it helps you forget about your problems**  **7 – Some other reason**  **9 - Prefer not to say** |

| DRUG USE | | | | |
| --- | --- | --- | --- | --- |
| **The next questions ask about your DRUG USE.** | **DRUG 1.** Have you ever used any of these drugs? Please answer YES or NO.  **(READ OUT LIST BELOW)** | **(ANY 'YES' in DRUG 1 ASK)  DRUG 2.** How old were you when you first used each of the drugs you mentioned? | **(ANY 'YES' in DRUG 1 ASK)  DRUG 3.** How often did you use it in THE last 12 months? **(SCROLL DOWN)** | **(ANY 'YES' in DRUG 1 ASK)  DRUG 4.** Has your drug use caused you any problems in the past year? (i.e.: with school, friends, health, police, parents) |
| **a.** Cannabis/Marijuana/ Yandi/Weed/Pot | **Drop down list**  **1 = Yes**  **[No = Leave Blank]** | **Drop down list**  **1 yr old**  **2 yr old**  **3 yr old**  **4 yr old**  **5 yr old**  **6 yr old**  **7 yr old**  **8 yr old**  **9 yr old**  **10 yr old**  **11 yr old**  **12 yr old**  **13 yr old**  **14 yr old**  **15 yr old**  **16 yr old**  **17 yr old**  **88 = Can't remember**  **99=Prefer not to say** | **Drop down list**  **1 = More than daily**  **2 = Daily**  **3 = 3 - 6 times per week**  **4 = 1 - 2 times per week**  **5 = 2 - 3 times per month**  **6 = Once per month**  **7 = Once or twice in last 12 months**  **8 = DID NOT USE in the last 12 months**  **9=Prefer not to say** | **Drop down list**  **0 = No**  **1 = Yes**  **9 = Prefer not to say** |
| **b.** Heroin | As above | As above | As above |  |
| **c.** Other opiates (eg. pethidine, morphine) | As above | As above | As above |  |
| **d.** Methadone/ Buprenorphine | As above | As above | As above |  |
| **e.** Subuxone/Subutex | As above | As above | As above |  |
| **f.** Amphetamine/speed | As above | As above | As above |  |
| **g.** Ice (Methamphetamines) | As above | As above | As above |  |
| **h.** Ecstasy/Designer drugs/MDMA | As above | As above | As above |  |
| **i.** LSD/Acid | As above | As above | As above |  |
| **j.** Tranquillisers/ Benzodiazepines | As above | As above | As above |  |
| **k.** Anaesthetics (eg. GHB, Ketamine) | As above | As above | As above |  |
| **l.** Volatile inhalants (eg. amyl nitrate) | As above | As above | As above |  |
| **m.** Volatile solvents (eg. petrol, glue) | As above | As above | As above |  |
| **n.** Steroids/Droids | As above | As above | As above |  |
| **o.** Other | *Type in answer* | As above | As above |  |
| **p.** Other | *Type in answer* | As above | As above |  |
| **q.** Other | *Type in answer* | As above | As above |  |

| INJECTING DRUG USE | | | | |
| --- | --- | --- | --- | --- |
| **Type of Drugs** | **(ANY 'YES' in DRUG 1 ASK)  INJ 1.** Have you ever injected any of these drugs you mentioned? **IF YES, which ones? (MAY HAVE MORE THAN ONE ANSWER)** | **INJ 2.** How old were you when you first injected drugs?  **(SCROLL DOWN FULL LIST)** | **INJ 3.** What was the last drug you injected? **(SCROLL DOWN FULL LIST)** | **INJ 4.** Have you ever injected in a juvenile detention, prison, or both? |
| **a.** Cannabis/Marijuana/ Yandi/Weed/Pot | **Drop down list**  **1 = Yes**  **[No = Leave Blank]** | **Drop down list**  **1 yr old**  **2 yr old**  **3 yr old**  **4 yr old**  **5 yr old**  **6 yr old**  **7 yr old**  **8 yr old**  **9 yr old**  **10 yr old**  **11 yr old**  **12 yr old**  **13 yr old**  **14 yr old**  **15 yr old**  **16 yr old**  **17 yr old**  **88 = Can't remember**  **99=Prefer not to say** | **Drop down list**  **1 = Yes**  **[No = Leave Blank]** | **Drop down list**  **0=No to all**  **1=Yes, in juvenile detention**  **2=Yes, in prison**  **3=Yes, in both places**  **9=Prefer not to say** |
| **b.** Heroin | As above |  | As above |  |
| **c.** Other opiates (eg. pethidine, morphine) | As above |  | As above |  |
| **d.** Methadone/ Buprenorphine | As above |  | As above |  |
| **e.** Subuxone/Subutex | As above |  | As above |  |
| **f.** Amphetamine/speed | As above |  | As above |  |
| **g.** Ice (Methamphetamines) | As above |  | As above |  |
| **h.** Ecstasy/Designer drugs/MDMA | As above |  | As above |  |
| **i.** LSD/Acid | As above |  | As above |  |
| **j.** Tranquillisers/ Benzodiazepines | As above |  | As above |  |
| **k.** Anaesthetics (eg. GHB, Ketamine) | As above |  | As above |  |
| **l.** Volatile inhalants (eg. amyl nitrate) | As above |  | As above |  |
| **m.** Volatile solvents (eg. petrol, glue) | As above |  | As above |  |
| **n.** Steroids/Droids | As above |  | As above |  |
| **o.** Other | Type in answer |  | As above |  |
| **p.** Other | Type in answer |  | As above |  |
| **q.** Other | Type in answer |  | As above |  |

| TOBACCO (SMHAC 2014) | |
| --- | --- |
| **The next questions ask about cigarette use** | |
| **YRB1.** Have you ever tried cigarette smoking, even one or two puffs? | **Drop down list**  **0=No (GO TO NEXT MODULE)**  **1=Yes**  **9=Prefer not to say (GO TO NEXT MODULE)** |
| **YRB2.** Was there ever a time in your life when you were smoking at least once per week? | **Drop down list**  **0=No**  **1=Yes**  **9=Prefer not to say** |
| **a.** How old were you when you first started smoking at least once per week? | **Drop down list**  **1 year old**  **2 years old**  **3 years old**  **4 years old**  **5 years old**  **6 years old**  **7 years old**  **8 years old**  **9 years old**  **10 years old**  **11 years old**  **12 years old**  **13 years old**  **14 years old**  **15 years old**  **16 years old**  **17 years old**  **99 = Prefer not to say** |
| **YRB4.** During the past 30 days, on how many days did you smoke cigarettes? | **Drop down list**  **1 = 0 days**  **2 = 1 or 2 days**  **3 = 3 to 5 days**  **4 = 6 to 9 days**  **5 = 10 to 19 days**  **6 = 20 to 29 days**  **7 = All 30 days**  **9 = Prefer not to say** |
| **YRB7.** Have you ever tried to quit smoking cigarettes? | **Drop down list**  **0=No**  **1=Yes**  **9=Prefer not to say** |

| SURVEY FEEDBACK | |
| --- | --- |
| **That’s the end of the study questions, but I’d like to ask some quick questions about the survey. We understand that the questions may have been difﬁcult to answer and don’t worry, we won’t make you do it again if you say it was.** | |
| **FEED1.** How embarrassing did you ﬁnd the questionnaire? | **Drop down list**  **1 = Extremely embarrassing**  **2 = Very embarrassing**  **3 = Quite embarrassing**  **4 = Slightly embarrassing**  **5 = Not at all embarrassing**  **9 = Prefer not to say** |
| **FEED2.** How many of your answers do you think were honest? **[This will not affect payment for the study]** | **Drop down list**  **1 = All**  **2 = Most**  **3 = About half**  **4 = A few**  **5 = None**  **9 = Prefer not to say** |
| **FEED3.** Would you be willing to do this survey again in the future? | **Drop down list**  **0 = No**  **1 = Yes**  **8 = Don't know/Unsure**  **9 = Prefer not to say** |
|  |  |
| **SURVEY END TIME:(24 HR)(HH:MM)** |  |
|  |  |
| **END SURVEY** | |
| **That’s it. Thank you for your help. Don’t forget to see the recruiter after this survey. If you would like to discuss any of the issues covered in this interview, please let the recruiter know. They will be providing you with information about the services available to you if you need to talk to someone or have any health concerns. Thank you once again.** | |

| TELEPHONE INTERVIEWER NOTES | **Type in your comments regarding this interview** |
| --- | --- |
|  |  |
|  |  |
|  |  |
|  |  |
|  |  |
|  |  |
|  |  |
|  |  |
|  |  |
|  |  |
|  |  |
|  |  |
|  |  |
